# Descriptive Epidemiology of Somatising Tendency: Findings from the CUPID Study

**DOI:** 10.1371/journal.pone.0153748

**Published:** 2016-04-29

**Authors:** Sergio Vargas-Prada, David Coggon, Georgia Ntani, Karen Walker-Bone, Keith T. Palmer, Vanda E. Felli, Raul Harari, Lope H. Barrero, Sarah A. Felknor, David Gimeno, Anna Cattrell, Matteo Bonzini, Eleni Solidaki, Eda Merisalu, Rima R. Habib, Farideh Sadeghian, M. Masood Kadir, Sudath S. P. Warnakulasuriya, Ko Matsudaira, Busisiwe Nyantumbu, Malcolm R. Sim, Helen Harcombe, Ken Cox, Leila M. M. Sarquis, Maria H. Marziale, Florencia Harari, Rocio Freire, Natalia Harari, Magda V. Monroy, Leonardo A. Quintana, Marianela Rojas, E. Clare Harris, Consol Serra, J. Miguel Martinez, George Delclos, Fernando G. Benavides, Michele Carugno, Marco M. Ferrario, Angela C. Pesatori, Leda Chatzi, Panos Bitsios, Manolis Kogevinas, Kristel Oha, Tiina Freimann, Ali Sadeghian, Roshini J. Peiris-John, Nalini Sathiakumar, A. Rajitha Wickremasinghe, Noriko Yoshimura, Helen L. Kelsall, Victor C. W. Hoe, Donna M. Urquhart, Sarah Derrett, David McBride, Peter Herbison, Andrew Gray, Eduardo J. Salazar Vega

**Affiliations:** 1 Center for Research in Occupational Health (CiSAL), Universitat Pompeu Fabra, Barcelona, Spain; 2 CIBER of Epidemiology and Public Health, Barcelona, Spain; 3 IMIM (Hospital del Mar Research Institute), Barcelona, Spain; 4 Medical Research Council Lifecourse Epidemiology Unit, University of Southampton, Southampton, United Kingdom; 5 Arthritis Research UK/MRC Centre for Musculoskeletal Health and Work, University of Southampton, Southampton, United Kingdom; 6 School of Nursing, University of São Paulo, São Paulo, Brazil; 7 Corporación para el Desarrollo de la Producción y el Medio Ambiente Laboral–IFA (Institute for the Development of Production and the Work Environment), Quito, Ecuador; 8 Department of Industrial Engineering, School of Engineering, Pontificia Universidad Javeriana, Bogotá, Colombia; 9 Southwest Center for Occupational and Environmental Health, The University of Texas Health Science Center at Houston School of Public Health, Houston, Texas, United States of America; 10 Center for Disease Control and Prevention/National Institute for Occupational Safety and Health, Atlanta, Georgia, United States of America; 11 North East London NHS Foundation Trust, Goodmayes Hospital, Ilford, United Kingdom; 12 Epidemiology and Preventive Medicine Research Center, University of Insubria, Varese, Italy; 13 Department of Social Medicine, Medical School, University of Crete, Heraklion, Greece; 14 Institute of Technology, Estonian University of Life Sciences, Tartu, Estonia; 15 Department of Environmental Health, Faculty of Health Sciences, American University of Beirut, Beirut, Lebanon; 16 Department of Occupational Health, School of Public Health, Shahroud University of Medical Sciences, Shahroud, Iran; 17 Department of Community Health Sciences, Aga Khan University, Karachi, Pakistan; 18 Department of Medical Education and Health Sciences, Faculty of Medical Sciences, University of Sri Jayewardenepura, Gangodawila, Nugegoda, Sri Lanka; 19 Department for Medical Research and Management for Musculoskeletal Pain, 22nd Century Medical and Research Center, Faculty of Medicine, The University of Tokyo Hospital, Tokyo, Japan; 20 National Institute for Occupational Health, National Health Laboratory Service, Johannesburg, South Africa; 21 Faculty of Health Sciences, University of Witwatersrand, Johannesburg, South Africa; 22 Department of Epidemiology and Preventive Medicine, School of Public Health and Preventive Medicine, Monash University, Melbourne, Victoria, Australia; 23 Department of Preventive and Social Medicine, University of Otago, Dunedin, New Zealand; 24 Federal University of Paraná, Curitiba-PR, Brazil; 25 School of Nursing of Ribeirão Preto, University of São Paulo, São Paulo, Brazil; 26 Program Health, Work and Environment in Central America, Institute for Studies on Toxic Substances (IRET), National University of Costa Rica, Heredia, Costa Rica; 27 Occupational Health Service, Parc de Salut MAR, Barcelona, Spain; 28 Servicio de Investigación y Análisis IT/EP, Departamento de Investigación y Análisis de Prestaciones, MC Mutual, Barcelona, Spain; 29 Department of Clinical Sciences and Community Health, Università degli Studi di Milano, Milan, Italy; 30 Fondazione Ca’ Granda Ospedale Maggiore Policlinico, Milan, Italy; 31 Department of Psychiatry, Medical School, University of Crete, Heraklion, Greece; 32 Centre for Research in Environmental Epidemiology (CREAL), Barcelona, Spain; 33 North Estonia Medical Centre, Tallinn, Estonia; 34 Tartu University Hospital, Tartu, Estonia; 35 Klinikum Leverkusen, Leverkusen, Germany; 36 Department of Physiology, Faculty of Medical Sciences, University of Sri Jayewardenepura, Gangodawila, Nugegoda, Sri Lanka; 37 Section of Epidemiology and Biostatistics, School of Population Health, Faculty of Medical and Health Sciences, University of Auckland, Auckland, New Zealand; 38 Department of Epidemiology, School of Public Health, University of Alabama at Birmingham, Birmingham, Alabama, United States of America; 39 Faculty of Medicine, University of Kalaniya, Kelaniya, Sri Lanka; 40 Department of Joint Disease Research, 22nd Century Medical and Research Center, University of Tokyo, Tokyo, Japan; 41 Centre for Occupational and Environmental Health, Department of Social and Preventive Medicine, Faculty of Medicine, University of Malaya, Kuala Lumpur, Malaysia; 42 Injury Prevention Research Unit, Department of Preventive and Social Medicine, University of Otago, Dunedin, New Zealand; 43 Health Safety and Environment Department, AkzoNobel, Houston, Texas, United States of America; Georgia Regents University, UNITED STATES

## Abstract

Somatising tendency, defined as a predisposition to worry about common somatic symptoms, is importantly associated with various aspects of health and health-related behaviour, including musculoskeletal pain and associated disability. To explore its epidemiological characteristics, and how it can be specified most efficiently, we analysed data from an international longitudinal study. A baseline questionnaire, which included questions from the Brief Symptom Inventory about seven common symptoms, was completed by 12,072 participants aged 20–59 from 46 occupational groups in 18 countries (response rate 70%). The seven symptoms were all mutually associated (odds ratios for pairwise associations 3.4 to 9.3), and each contributed to a measure of somatising tendency that exhibited an exposure-response relationship both with multi-site pain (prevalence rate ratios up to six), and also with sickness absence for non-musculoskeletal reasons. In most participants, the level of somatising tendency was little changed when reassessed after a mean interval of 14 months (75% having a change of 0 or 1 in their symptom count), although the specific symptoms reported at follow-up often differed from those at baseline. Somatising tendency was more common in women than men, especially at older ages, and varied markedly across the 46 occupational groups studied, with higher rates in South and Central America. It was weakly associated with smoking, but not with level of education. Our study supports the use of questions from the Brief Symptom Inventory as a method for measuring somatising tendency, and suggests that in adults of working age, it is a fairly stable trait.

## Introduction

Somatising tendency is a predisposition to be unusually aware of, and to worry about, common somatic symptoms [1]. It can be measured through instruments such as the Somatic Symptom Scale [2], the Modified Somatic Perception Questionnaire [3], and a scale derived from the Brief Symptom Inventory [4], and is associated with various aspects of health and health-related behaviour. These include musculoskeletal pain [5–8], especially at multiple sites [9–15], sickness absence from work [16,17], medical consultation [18] and dissatisfaction with medical care [18]. Moreover, the relationship to pain has been observed in longitudinal as well as cross-sectional studies, indicating that tendency to somatise predicts, and is not simply a consequence of, other aspects of health [4–8,14,19,20].

In view of its potential to explain differences in health and behaviour, it is important to understand better the nature of somatising tendency and its descriptive epidemiology. It would be helpful to establish: i) how it can be assessed most efficiently (avoiding redundant information); ii) whether it should be viewed as a long-term trait or a variable state; iii) how it relates to personal characteristics such as sex, age and level of education; and iv) whether it varies importantly between countries and cultures. To explore these questions, we used data from the Cultural and Psychosocial Influences on Disability (CUPID) study, a large international longitudinal investigation of musculoskeletal pain and its determinants [21].

## Methods

The design of the CUPID study and its methods of data collection have been reported in detail elsewhere [21]. In brief, the study sample comprised a total of 12,426 participants aged 20–59 years from 47 occupational groups in 18 countries. The occupational groups fell into three broad categories–nurses (including nursing assistants), office staff who regularly used computers, and other workers (mainly manual employees carrying out repetitive tasks with their hands or arms). Each of the 12,426 participants completed a baseline questionnaire (either by self-administration, or in some occupational groups at interview), representing an overall response rate of approximately 70% among those who were eligible for inclusion [21]. After a mean interval of 14 months (range 3–35 months, 84% within 11–19 months), participants in 45 of the 47 occupational groups (n = 11,992) were asked to complete a shorter follow-up questionnaire, and responses were obtained from 9,305 (78%).

The questionnaires were originally drafted in English, and were then translated into local languages where necessary, accuracy being checked by independent back-translation. Among other things, the baseline questionnaire covered sex; age; age of completing full-time education; smoking habits; experience of pain in the past month at each of ten anatomical sites (low back; neck; and right and left shoulder, elbow, wrist/hand and knee) illustrated by diagrams; duration of sickness absence in the past 12 months because of illness other than a problem with the back, neck, upper limb or knees; and somatising tendency.

Somatising tendency was assessed through questions taken from the Brief Symptom Inventory [4], which asked how distressed or bothered (on a five-point ordinal scale from “not at all” to “extremely”) the participant had been during the past seven days by each of: faintness or dizziness, pains in the heart or chest, nausea or upset stomach, trouble getting breath, numbness or tingling in parts of the body, feeling weak in parts of the body, and hot or cold spells. A symptom was deemed to occur if it was at least moderately distressing (i.e. in the highest three of the five levels). The same questions were asked both at baseline and at follow-up.

Statistical analysis was carried out with Stata (StataCorp LP 2012, Stata Statistical Software: Release 12.1, College Station, Texas, USA). Pairwise associations between somatic symptoms at baseline were summarised by odds ratios adjusted for sex and age, as were those between symptoms at baseline and at follow-up.

To explore the clustering of symptoms within individuals, we compared the frequency with which a given number of symptoms was reported with the frequency that would have been expected given the overall prevalence of each symptom, and assuming that their occurrence was mutually independent (for example, that experience of chest pain did not make it more or less likely that an individual would suffer from numbness or tingling). Within each of eight strata defined by combinations of sex and 10-year age band, the expected frequency of each possible combination of symptoms was calculated. These expected frequencies were then summed for combinations representing the same total number of symptoms, and the totals further summed across the eight strata to give the overall number of participants who would be expected to have that number of symptoms.

The relationship of different counts of somatic symptoms to multi-site pain in the past month (defined as pain at ≥4 of 10 anatomical sites) was assessed by Poisson regression, with adjustment for sex and age. Possible clustering of the pain outcome by occupational group was taken into account by random intercept, multi-level modelling. Associations were summarised by prevalence rate ratios (PRRs) with 95% confidence intervals (95%CIs) based on robust standard errors. To explore whether somatising tendency could be adequately characterised without asking about all seven symptoms, we repeated the analysis, excluding data on specific symptoms in turn, and compared population attributable fractions (PAFs–defined as the proportions of cases in a population that would be eliminated if all people had the same risk as those in the reference category). Confidence intervals for PAFs were derived by bootstrapping. To check that findings were not specific to associations with multi-site pain, we repeated the analyses with an alternative outcome–absence from work for >5 days in the past year for reasons other than a problem with the back, neck, upper limb or knees.

We used simple descriptive statistics to summarise changes in the occurrence of somatic symptoms from baseline to follow-up, and the prevalence of symptoms by occupational group. To test whether there was greater similarity in the occurrence of symptoms within as compared to between countries, we calculated the intra-class correlation coefficient (ICC) for the mean numbers of symptoms by occupational group.

We also investigated the possibility that some occupational groups might have a different profile of somatic symptoms from others. For each combination of occupational group and symptom, we compared the number of participants in the group who reported the symptom, with the number that would have been expected to report it if, after allowance for sex and age, the frequency of the symptom as a proportion of all symptom reports in the occupational group were the same as that in the full study sample. A ratio of observed to expected greater than one was an indication that the occupational group experienced the symptom more often than would have been expected, given their overall tendency to somatise.

Finally, we used Poisson regression to assess the (mutually adjusted) cross-sectional associations of somatising tendency at baseline (defined as report of ≥3 somatic symptoms) with possible risk factors (sex, age, smoking habits and age finished full-time education). Again random intercept modelling was used to allow for possible clustering by occupational group.

Ethical approval for the study was provided by the relevant research ethics committee or institutional review board in each participating country (S1 Appendix).

## Results

In one occupational group (office workers in Colombia), one of the questions about somatic symptoms had been omitted. Complete data on somatic symptoms at baseline were available for 12,072 men and women from the remaining 46 occupational groups (98% of all participants from those groups). Table 1 shows the prevalence of each symptom by sex and age. Among men, the prevalence of all symptoms except numbness or tingling was highest in the youngest age group (20–29 years). Women reported each of the seven symptoms more frequently than men, and particularly nausea or upset stomach, hot or cold spells (especially at older ages), and numbness or tingling (again more at older ages). Moreover, in contrast to men, the only symptoms that were most common at age 20–29 years were faintness or dizziness and nausea or upset stomach. In view of these differences, all subsequent analyses were adjusted for sex and age.

**Table 1:** Baseline prevalence (%) of distressing somatic symptoms in past 7 days by sex and age.

Symptom	Men				Women			
	20–29 years	30–39 years	40–49 years	50–59 years	20–29 years	30–39 years	40–49 years	50–59 years
	(N = 1,056)	(N = 1,379)	(N = 1,170)	(N = 641)	(N = 1,954)	(N = 2,487)	(N = 2,172)	(N = 1,213)
Faintness or dizziness	8.0 (85)	7.2 (99)	6.2 (73)	4.8 (31)	17.3 (339)	15.9 (395)	15.1 (328)	12.0 (146)
Pains in heart or chest	10.1 (107)	7.0 (97)	5.8 (68)	5.8 (37)	9.6 (188)	10.0 (248)	12.4 (269)	10.8 (131)
Nausea or upset stomach	16.3 (172)	12.8 (177)	11.4 (133)	9.7 (62)	27.0 (528)	25.3 (630)	22.9 (497)	18.3 (222)
Trouble getting breath	7.1 (75)	5.7 (79)	5.6 (65)	5.5 (35)	10.1 (197)	10.0 (149)	10.6 (230)	10.2 (124)
Hot or cold spells	16.7 (176)	11.8 (163)	10.8 (126)	9.8 (63)	21.6 (423)	21.5 (535)	26.9 (584)	35.1 (426)
Feeling weak in parts of your body	21.3 (225)	17.3 (238)	18.9 (221)	18.6 (119)	26.7 (522)	30.7 (763)	30.9 (671)	28.3 (343)
Numbness or tingling in parts of your body	14.8 (156)	11.6 (160)	16.0 (187)	14.8 (95)	17.2 (336)	25.0 (621)	30.8 (670)	29.6 (359)

Figures in brackets are the numbers of participants with the relevant symptom

Table 2 summarises the associations between pairs of somatic symptoms at baseline. The strongest associations were for pain in the heart or chest with trouble getting breath (OR 9.3), and feeling weak in parts of the body with numbness or tingling in parts of the body (OR 7.9). However, all symptoms were associated with each other, the lowest odds ratio being 3.4.

**Table 2:** Pairwise associations between specific somatic symptoms at baseline.

Symptom at baseline	Faintness or dizziness	Pains in heart or chest	Nausea or upset stomach	Trouble getting breath	Hot or cold spells	Feeling weak in parts of body
Pains in heart or chest	6.6 (492)					
Nausea or upset stomach	5.8 (802)	4.5 (587)				
Trouble getting breath	5.6 (429)	9.3 (450)	4.4 (547)			
Hot or cold spells	4.0 (706)	3.4 (548)	3.8 (1,050)	4.1 (544)		
Feeling weak in parts of body	5.1 (874)	4.7 (681)	3.9 (1,249)	4.9 (653)	4.9 (1,365)	
Numbness or tingling in parts of body	3.9 (705)	4.4 (607)	3.4 (1,032)	4.7 (587)	3.6 (1,113)	7.9 (1,625)

Associations are summarised by odds ratios adjusted for sex and age, with the number of participants reporting both symptoms in brackets

Table 3 compares the frequency with which specified numbers of symptoms were reported and the frequency that would have been expected had the occurrence of each symptom been statistically independent. More participants than expected reported no symptoms at all (6,016 vs. 3,433). However, there were fewer than expected with 1–3 symptoms. The ratio of observed to expected numbers then increased progressively for report of larger numbers of symptoms, rising from 2.75 for four symptoms to 2000 for all seven symptoms.

**Table 3:** Observed and expected frequency of multiple somatic symptoms and associations with multi-site pain.

Number of somatic symptoms	Observed number of subjects	Expected number of subjects^a^	Ratio of observed to expected	Association with pain at ≥4 vs. 0 anatomical sites					
				Number with no pain	Number with pain at ≥4 sites	PRR^b^	(95%CI)	PAF^c^ (%)	(95%CI)
0	6,016	3,433	1.75	3,125	374	1			
1	2,312	4,546	0.51	779	291	2.3	(2.0–2.7)	8.9	(6.8–11.0)
2	1,551	2,817	0.55	336	342	4.0	(3.3–4.8)	13.7	(11.5–15.8)
3	944	1,020	0.93	149	295	5.0	(4.1–6.1)	12.6	(10.7–14.6)
4	618	224.6	2.75	86	226	5.1	(4.1–6.5)	9.7	(7.6–11.7)
5	326	29.26	11.1	30	164	5.9	(4.8–7.4)	7.3	(5.5–9.1)
6	185	2.058	89.9	14	104	6.0	(4.6–7.7)	4.6	(3.2–6.0)
7	120	0.060	2000	15	78	5.8	(4.6–7.2)	3.4	(2.2–4.7)
≥1	6,056	8,639.18	0.70	1,409	1,500	3.8	(3.2–4.5)	59.0	(53.5–64.5)
≥3	2,193	1,276.23	1.72	294	867	5.6	(4.4–7.0)	37.9	(30.9–44.9)

^a^ Expected number given the overall prevalence of each symptom, and assuming no association between the occurrence of one symptom and another after allowance for sex and age (in four 10-year strata)

^b^ Prevalence rate ratio adjusted for sex and age (in four 10-year strata)

^c^ Population attributable fraction

Table 3 also shows the associations between the number of somatic symptoms and report of pain at ≥4 of 10 anatomical sites. Relative to no somatic symptoms, PRRs for multisite pain increased progressively from 2.3 (95%CI 2.0–2.7) for one somatic symptom to 5.9 (95%CI 4.8–7.4) for five somatic symptoms, and then remained at a similar level for six and seven symptoms. The right-hand columns of the table give the corresponding population attributable fractions (PAFs) and their 95% CIs. Overall, report of at least one somatic symptom accounted for 59.0% of the cases of multi-site pain in the study sample.

To explore whether information about any of the somatic symptoms was effectively redundant, we repeated the analysis of associations with multi-site pain excluding each of the seven symptoms in turn (Table 4). In each case, the PAF for multi-site pain that was associated with report of at least one of the remaining somatic symptoms was lower than in the analysis that included all somatic symptoms (53.2% to 58.6% vs. 59.0%), indicating that each symptom added to the characterisation of somatising tendency, although an index based on only six of the seven symptoms would still work well.

**Table 4:** Associations of multiple somatic symptoms with multi-site pain when one of the seven somatic symptoms was ignored.

Somatic symptom disregarded	Number of somatic symptoms														
	1		2		3		4		5		6		≥1 somatic symptom		
	PRR^a^	(95%CI)	PRR^a^	(95%CI)	PRR^a^	(95%CI)	PRR^a^	(95%CI)	PRR^a^	(95%CI)	PRR^a^	(95%CI)	PRR^a^	(95%CI)	PAF^b^ (%)
Faintness or dizziness	2.4	(2.1–2.8)	3.9	(3.3–4.7)	5.1	(4.2–6.3)	5.2	(4.2–6.5)	5.8	(4.6–7.5)	5.7	(4.6–7.1)	3.7	(3.2–4.4)	57.6
Pains in heart or chest	2.5	(2.1–2.9)	4.1	(3.4–5.0)	5.0	(4.1–6.2)	5.4	(4.3–6.8)	5.8	(4.5–7.4)	5.9	(4.7–7.3)	3.8	(3.2, 4.5)	58.6
Nausea or upset stomach	2.5	(2.1–3.0)	4.2	(3.5–5.0)	4.8	(3.9–5.9)	5.5	(4.4–6.8)	5.6	(4.4–7.1)	5.4	(4.4–6.7)	3.8	(3.2–4.4)	57.0
Trouble getting breath	2.4	(2.1–2.7)	4.1	(3.4–5.0)	5.0	(4.1–6.0)	5.3	(4.3–6.6)	6.1	(4.8–7.7)	5.8	(4.6–7.2)	3.8	(3.2–4.4)	58.6
Hot or cold spells	2.5	(2.2–3.0)	4.3	(3.6–5.2)	4.8	(3.8–6.0)	5.3	(4.2–6.7)	5.6	(4.4–7.1)	5.1	(4.0–6.5)	3.8	(3.2–4.5)	56.7
Feeling weak in parts of your body	2.5	(2.1–2.9)	3.7	(3.1–4.3)	4.3	(3.6–5.2)	4.7	(3.8–5.8)	5.1	(4.1–6.5)	4.8	(3.9–5.9)	3.3	(2.9–3.9)	53.2
Numbness or tingling in parts of your body	2.5	(2.2–2.9)	3.8	(3.2–4.6)	4.2	(3.5–5.1)	4.8	(3.9–6.0)	4.8	(3.9–6.0)	5.1	(4.0–6.3)	3.4	(3.0–4.0)	54.4

^a^ Prevalence rate ratio, adjusted for sex and age (in four 10-year strata), for pain at ≥4 vs. 0 anatomical sites in participants with the specified number of somatic symptoms compared with no somatic symptoms. The specified number of symptoms was from the total of six that remained when the symptom in the left-hand column was disregarded.

^b^ Population attributable fraction

To check that these patterns of association were not specific to pain outcomes, we repeated the analyses for Tables 3 and 4, taking as an alternative outcome sickness absence in the past 12 months for non-musculoskeletal reasons. In the analysis that included all seven somatic symptoms, PRRs rose progressively from 1.4 (95%CI 1.1–1.7) for report of one symptom to 3.2 (95%CI 2.4–4.2) for report of seven symptoms, and the PAF for report of at least one somatic symptom was 30.4% (Table 5). The PAFs when single somatic symptoms were disregarded ranged from 27.1% to 30.4% (Table 6).

**Table 5:** Associations between number of somatic symptoms and sickness absence for >5 days in past 12 months for non-musculoskeletal reasons.

Number of somatic symptoms	Duration of sickness absence in past 12 months for reasons other than musculoskeletal pain					
	None	****>5 days****				
	****N****	****N****	****PRR****^a^	****(95%CI)****	****PAF****^b^ ****(%)****	****(95%CI)****
0	3,982	408	**1**			
1	1,377	225	1.4	(1.1–1.7)	5.1	(2.3–7.9)
2	863	200	1.8	(1.6–2.2)	7.7	(5.8–9.6)
3	453	135	2.3	(1.8–2.8)	6.3	(4.4–8.2)
4	266	104	2.8	(2.2–3.4)	5.5	(3.5–7.6)
5	154	60	2.7	(2.1–3.5)	3.2	(1.7–4.7)
6	70	34	2.9	(2.0–4.1)	1.9	(0.8–2.9)
7	44	28	3.2	(2.4–4.2)	1.6	(0.7–2.5)
≥1	3,227	786	1.9	(1.6–2.2)	30.4	(23.9–36.9)
≥3	987	361	2.5	(2.1–3.1)	18.4	(10.9–25.8)

^a^ Prevalence rate ratio relative to no sickness absence in past 12 months for non-musculoskeletal reasons, adjusted for sex and age (in four 10-year strata)

^b^ Population attributable fraction

**Table 6:** Associations of multiple somatic symptoms with sickness absence for >5 days in past 12 months for non-musculoskeletal reasons when one of the seven somatic symptoms was ignored.

****Somatic symptom disregarded****	****Number of somatic symptoms****														
	****1****		****2****		****3****		****4****		****5****		****6****		****≥1 somatic symptom****		
	****PRR****^a^	****(95%CI)****	****PRR****^a^	****(95%CI)****	****PRR****^a^	****(95%CI)****	****PRR****^a^	****(95%CI)****	****PRR****^a^	****(95%CI)****	****PRR****^a^	****(95%CI)****	****PRR****^a^	****(95%CI)****	****PAF****^b^ ****(%)****
Faintness or dizziness	1.4	(1.2–1.6)	2.0	(1.7–2.3)	2.4	(1.9–3.0)	2.4	(1.9–3.1)	2.8	(2.1–3.8)	3.0	(2.3–3.9)	1.8	(1.6–2.1)	29.3
Pains in heart or chest	1.4	(1.2–1.7)	1.8	(1.6–2.1)	2.3	(1.9–2.8)	2.8	(2.2–3.5)	2.6	(2.0–3.3)	3.6	(2.7–4.9)	1.8	(1.6–2.2)	29.6
Nausea or upset stomach	1.4	(1.2–1.7)	1.9	(1.6–2.2)	2.4	(2.0–2.9)	2.7	(2.1–3.4)	2.6	(1.9–3.6)	2.9	(2.1–3.9)	1.8	(1.6–2.1)	28.0
Trouble getting breath	1.4	(1.2–1.6)	1.9	(1.6–2.2)	2.1	(1.7–2.7)	2.7	(2.2–3.3)	2.9	(2.1–3.8)	2.9	(2.1–3.9)	1.8	(1.6–2.1)	29.4
Hot or cold spells	1.6	(1.3–1.8)	2.0	(1.7–2.3)	2.5	(2.0–3.1)	2.7	(2.1–3.5)	2.9	(2.2–3.9)	2.9	(2.2–3.8)	2.0	(1.7–2.3)	30.4
Feeling weak in parts of your body	1.4	(1.2–1.6)	1.9	(1.6–2.3)	2.4	(2.0–3.0)	2.9	(2.3–3.6)	3.0	(2.1–4.1)	2.9	(2.2–3.8)	1.8	(1.6–2.1)	27.1
Numbness or tingling in parts of your body	1.5	(1.2–1.7)	2.0	(1.7–2.3)	2.7	(2.3–3.3)	2.6	(2.1–3.3)	2.5	(1.7–3.7)	3.1	(2.4–4.0)	1.9	(1.6–2.2)	29.5

^a^ Prevalence rate ratio, adjusted for sex and age (in four 10-year strata), for sickness absence in the past 12 months for non-musculoskeletal reasons vs. 0 days of sickness absence in participants with the specified number of somatic symptoms compared with no somatic symptoms. The specified number of symptoms was from the total of six that remained when the symptom in the left-hand column was disregarded.

^b^ Population attributable fraction

Complete information about somatic symptoms at follow-up was available for 8,856 (73%) of the participants who provided satisfactory information at baseline, the follow-up rate being similar in those who initially did and did not have symptoms. Table 7 shows the number of somatic symptoms that they reported at follow-up, according to the number that were present at baseline. In general, participants reported similar numbers of symptoms at follow-up as at baseline, 6,677 (75%) having a change of zero or one in their symptom count. There were, however, notable exceptions. In particular, seven participants went from zero symptoms at baseline to seven at follow-up, and 19 changed to the same extent in the reverse direction. More detailed examination of the questionnaire responses for these 26 individuals indicated that for the most part, the changes represented substantial differences in the levels of distress reported from individual symptoms, and not simply a shift from their being “a little bit” to “moderately” distressing.

**Table 7:** Number of somatic symptoms reported at follow-up according to number of somatic symptoms reported at baseline.

****Number of symptoms at baseline****	****Number of symptoms at follow-up****							
	****0****	****1****	****2****	****3****	****4****	****5****	****6****	****7****
0	3,329	622	235	107	49	18	10	7
1	885	479	216	108	47	15	2	0
2	452	293	217	101	52	21	12	2
3	230	151	137	98	54	16	14	4
4	141	71	81	75	47	29	19	5
5	51	31	40	41	26	17	7	5
6	20	12	18	16	13	16	9	5
7	19	13	5	10	5	11	8	7

Analysis was restricted to the 8,856 participants who provided complete information about somatic symptoms at both baseline and follow-up.

The 8,856 participants who provided complete information at both time-points reported a total of 10,326 somatic symptoms at baseline. Of these specific symptoms, 3,733 (36%) were again reported at follow-up, while 6,593 (64%) had resolved. On the other hand, 4,123 (52%) of a total of 7,856 symptoms at follow-up were new since baseline. Table 8 summarises the pairwise associations between specific somatic symptoms at baseline and at follow-up. The highest odds ratios (3.6 to 6.6) were for continuing presence of the same symptom at follow-up as at baseline, but all odds ratios were ≥1.6, and most were ≥2.0.

**Table 8:** Pairwise associations between specific somatic symptoms at baseline and at follow-up.

Symptom at baseline	Symptom at follow-up						
	Faintness or dizziness	Pains in heart or chest	Nausea or upset stomach	Trouble getting breath	Hot or cold spells	Feeling weak in parts of body	Numbness or tingling in parts of body
	(n = 741)	(n = 542)	(n = 1,254)	(n = 558)	(n = 1,445)	(n = 1,778)	(n = 1,538)
Faintness or dizziness (n = 1,030)	5.0 (293)	2.6 (149)	2.4 (295)	2.1 (133)	2.1 (304)	2.3 (377)	2.5 (351)
Pains in heart or chest (n = 753)	2.6 (155)	5.6 (182)	2.0 (196)	2.6 (115)	1.8 (216)	2.0 (264)	2.1 (252)
Nausea or upset stomach (n = 1,719)	2.6 (287)	2.0 (190)	3.6 (545)	1.7 (179)	1.6 (421)	2.0 (556)	1.7 (462)
Trouble getting breath (n = 748)	2.6 (148)	3.2 (132)	2.1 (202)	6.6 (199)	1.8 (215)	2.3 (286)	2.2 (253)
Hot or cold spells (n = 1,833)	2.2 (285)	2.1 (219)	2.0 (438)	1.9 (208)	3.9 (709)	2.1 (620)	2.0 (557)
Feeling weak in parts of body (n = 2,317)	2.6 (361)	2.4 (263)	2.1 (538)	2.2 (265)	2.0 (611)	4.3 (983)	2.9 (760)
Numbness or tingling in parts of body (n = 1,926)	2.5 (306)	2.7 (249)	2.0 (452)	1.9 (220)	1.9 (534)	2.7 (729)	5.1 (822)

Associations are summarised by odds ratios adjusted for sex and age (in 10-year strata), with the number of participants reporting both symptoms in brackets. Analysis was restricted to the 8,856 participants who provided complete information about somatic symptoms at both baseline and follow-up.

**Fig 1:**
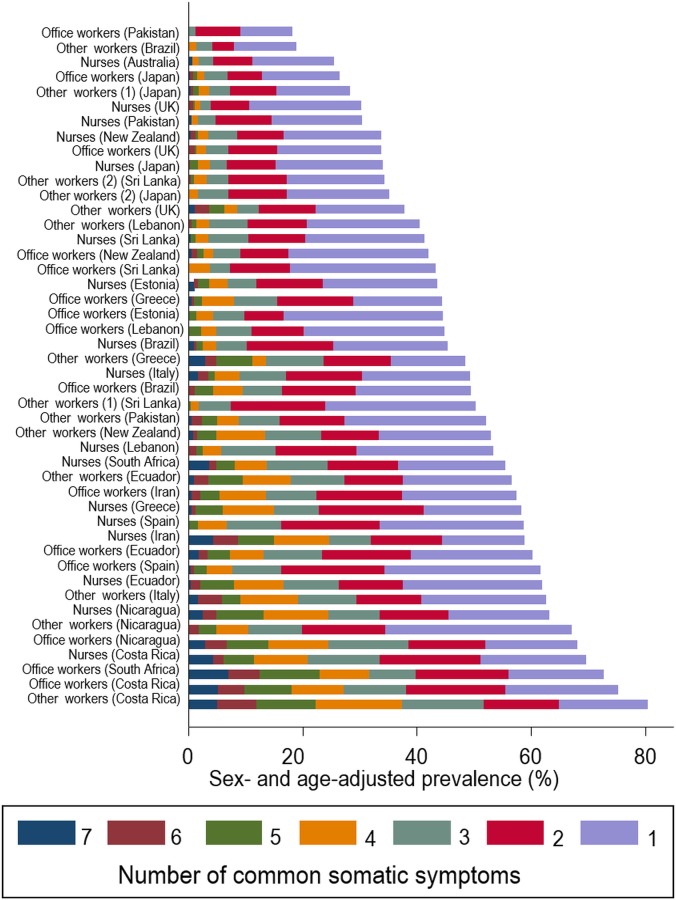
Frequency of somatic symptoms by occupational group.

Fig 1 shows the prevalence of different numbers of somatic symptoms at baseline by occupational group. There was major variation between the groups–for example, the prevalence of ≥3 somatic symptoms ranged from 1.3% among office workers in Pakistan and 4.2% in sugar cane cutters in Brazil to 38.1% in office workers in Costa Rica and 51.8% in manual workers in Costa Rica. Apart from the Brazilian sugar cane cutters, rates in South and Central America were all relatively high. The mean numbers of symptoms by occupational group showed greater similarity within than between countries (ICC = 15%). However, there was no consistent pattern by type of occupation (nurse, office worker or other).

To explore whether any occupational groups displayed a distinct profile of somatic symptoms, we compared the proportionate frequency of specific symptoms after standardisation for sex and age. The standardised proportions ranged from 0 for hot or cold spells in Brazilian sugar cane cutters and 0.15 for hot or cold spells in Sri Lankan postal workers to 1.98 for nausea or upset stomach in Japanese sales personnel and 2.10 for hot or cold spells in Pakistani postal workers. However, the large majority were between 0.67 and 1.5. The most salient patterns by country were high ratios for trouble getting breath in Brazil (1.28–1.56); low ratios for hot or cold spells in Greece (0.37–0.73); high ratios for faintness or dizziness (1.73 and 1.75) and low ratios for feeling weak (0.31 and 0.49) in Estonia; low ratios for pains in the heart or chest in Lebanon (0.42–0.63); low ratios for each of faintness or dizziness (0.58–0.72), pains in the heart or chest (0.23–0.78) and trouble getting breath (0.28–0.80), and high ratios for hot or cold spells (1.44–2.10) in Pakistan; high ratios for nausea or upset stomach (1.17–1.98) and low ratios for trouble getting breath (0.24–0.53) in Japan; and high ratios for pains in the heart or chest in South Africa (1.57 and 1.77). Further details are given in Table 9.

**Table 9:** Standardised proportions of specific symptoms by occupational group.

Occupational group	Symptom						
	Faintness or dizziness	Pains in heart or chest	Nausea or upset stomach	Trouble getting breath	Hot or cold spells	Feeling weak in parts of your body	Numbness or tingling in parts of your body
**Brazil**							
Nurses	0.58	1.03	0.77	1.28	0.80	1.06	1.43
Office workers	0.60	1.43	0.69	1.56	0.89	0.94	1.21
Other workers	1.32	1.27	0.72	1.54	0.00	0.83	1.89
**Ecuador**							
Nurses	1.13	0.94	1.00	0.63	1.29	0.96	0.84
Office workers	0.63	1.17	1.08	0.87	1.05	1.02	1.05
Other workers	1.06	1.37	0.91	1.12	1.11	0.98	0.75
**Costa Rica**							
Nurses	0.56	0.94	1.01	0.92	1.12	1.02	1.16
Office workers	0.71	1.02	1.12	1.16	1.15	0.83	1.04
Other workers	0.96	1.18	0.88	1.03	0.90	0.95	1.20
**Nicaragua**							
Nurses	0.78	0.80	1.03	0.89	1.12	0.91	1.19
Office workers	0.70	1.04	0.89	1.17	1.12	0.95	1.15
Other workers	0.53	0.54	0.83	1.52	1.23	1.07	1.16
**UK**							
Nurses	0.92	1.28	1.13	0.75	1.18	0.90	0.86
Office workers	1.05	1.06	1.05	1.08	1.05	0.96	0.88
Other workers	1.31	1.14	1.00	1.12	1.07	0.95	0.75
**Spain**							
Nurses	0.73	0.52	0.93	0.76	0.64	1.56	1.23
Office workers	0.66	0.56	0.89	0.88	1.17	1.24	1.09
**Italy**							
Nurses	1.16	1.06	1.24	1.18	0.87	0.88	0.86
Other workers	0.94	0.87	0.96	1.44	0.83	1.05	1.06
**Greece**							
Nurses	1.22	0.74	0.98	0.93	0.37	1.20	1.34
Office workers	1.08	1.18	0.96	0.63	0.73	1.11	1.19
Other workers	1.39	0.91	0.81	1.45	0.58	1.10	1.05
**Estonia**							
Nurses	1.75	1.77	0.84	1.15	1.03	0.49	0.90
Office workers	1.73	1.29	0.91	1.96	0.95	0.31	1.01
**Lebanon**							
Nurses	1.07	0.42	1.35	1.14	0.63	1.13	0.96
Office workers	0.71	0.48	1.25	1.68	0.57	1.15	1.11
Other workers	0.95	0.63	0.70	1.44	1.21	0.89	1.29
**Iran**							
Nurses	1.50	1.32	0.84	1.23	0.90	1.01	0.68
Office workers	1.86	1.05	0.38	0.74	1.40	1.00	0.79
**Pakistan**							
Nurses	0.58	0.36	0.50	0.36	1.44	1.47	1.54
Office workers	0.64	0.23	0.83	0.28	1.86	1.43	0.68
Other workers	0.72	0.78	0.72	0.80	2.10	1.09	0.57
**Sri Lanka**							
Nurses	1.07	0.67	1.08	1.51	1.41	0.64	0.80
Office workers	0.76	1.11	1.01	0.87	1.33	0.72	1.18
Other workers (1)	0.83	1.26	0.80	0.99	0.15	1.40	1.20
Other workers (2)	0.82	1.59	1.05	0.79	1.15	0.95	0.79
**Japan**							
Nurses	1.38	0.87	1.56	0.53	0.90	0.85	0.66
Office workers	1.70	0.80	1.25	0.24	1.14	0.84	0.88
Other workers (1)	1.34	0.80	1.17	0.42	0.94	1.13	0.91
Other workers (2)	1.13	0.68	1.98	0.35	1.05	0.84	0.56
**South Africa**							
Nurses	1.20	1.57	0.83	1.16	1.06	0.83	0.88
Office workers	1.04	1.77	1.06	1.22	0.98	0.71	0.84
**Australia**							
Nurses	1.08	0.49	1.33	1.03	0.87	1.07	0.92
**New Zealand**							
Nurses	0.90	1.09	1.15	0.49	1.21	0.97	0.91
Office workers	0.62	0.58	1.36	0.83	1.12	1.05	0.97
Other workers	0.84	0.85	1.05	0.92	1.08	1.15	0.88

Standardised proportions were calculated as *O*/∑_*i*_(*n*_*i*_ * *S*_*i*_/*N*_*i*_) where *O* was the observed frequency of the specified symptom in the occupational group, and within the i^th^ of 8 strata of sex and 10-year age band, *n*_*i*_ was the total number of symptom reports (any of the seven symptoms) in the occupational group, *S*_*i*_ was the number of reports of the specified symptom in all occupational groups combined, and *N*_*i*_ was the total number of symptom reports (any of the seven symptoms) in all occupational groups combined.

In a mutually adjusted analysis of the cross-sectional association between personal characteristics and somatising tendency (pragmatically specified as report of ≥3 somatic symptoms), there was a significantly elevated risk with female sex (PRR 1.8, 95%CI 1.5–2.1), and a weak but significant relationship to smoking habits (PRRs of 1.3 and 1.2 for current and ex- as compared with non-smokers). However, there was no association with age of finishing full-time education (data not shown).

## Discussion

Within our large study sample, the seven somatic complaints that we examined were all mutually associated, such that report of multiple symptoms was much more frequent than would have been expected had their occurrence been unrelated. However, no cut-point in the number of reported symptoms distinguished unequivocally between people with and without a somatising syndrome. Rather, there appeared to be a gradation in degrees of tendency to somatise. In most individuals, the level of somatising tendency (as assessed by the questionnaire) was little changed after a follow-up interval of approximately 14 months, although the specific symptoms reported at follow-up often differed from those at baseline. Tendency to somatise was more common in women than men, especially at older ages, and after allowance for sex and age, it varied markedly across the 46 occupational groups studied, with greater similarities within than between countries. It was weakly associated with smoking, but not with level of education.

As well as the size, geographical spread and cultural diversity of the study sample, our investigation benefitted from high response rates. However, it was limited to adults of working age, and the findings cannot necessarily be extrapolated to other age groups. It was also restricted to selected occupational groups, although apart perhaps from sugar cane cutters in Brazil, it seems unlikely that these will have been highly unrepresentative of the wider working populations in participating countries.

Somatising tendency was assessed through seven questions taken from the Brief Symptom Inventory, which has been established as a valid and reliable instrument [22] with the ability to predict future health outcomes in longitudinal investigations [6–8]. Moreover, where it was necessary to translate the questionnaire into local languages, care was taken to check accuracy through independent back-translation. Nevertheless, it is possible that symptoms were understood differently across varied cultural settings. Such variation may have contributed to differences in prevalence between countries, but would not explain associations with other variables measured at individual level in analyses that adjusted for possible clustering by job group.

We did not have information about personality traits or about other medical conditions such as cancer, which may have caused some of the symptoms that distressed participants. However, since our study sample comprised adults in active employment, the prevalence of serious co-morbidity will have been low, and should not have impacted importantly on our conclusions.

Understanding of terms for pain may have varied between participants speaking different languages, but the anatomical location of symptoms should have been unambiguous, since it was defined pictorially. Errors of interpretation are less likely to have occurred for other variables such as history of sickness absence, smoking habits and educational level, although they may have been liable to inaccurate recall. Provided inaccuracies were not differential in relation to somatising tendency, any resultant bias in associations with somatising tendency will have been towards the null.

Much of the literature on somatisation has focused on medically unexplained somatic symptoms as a reason for presentation to medical care, and a manifestation of hidden psychiatric morbidity. As defined in the tenth revision of the International Classification of Diseases (ICD10), somatisation disorder is generally infrequent, with prevalence rates among adults aged 18–65 years in a cross-cultural study of 14 countries mostly less than 2% [23]. However, our interest was in the wider spectrum of distress from common somatic symptoms, not necessarily leading to medical consultation of themselves, but collectively associated with other aspects of health and health-related behaviour. By limiting our enquiry to symptoms in the past week, we reduced the potential for errors in recall, which can be a problem when longer periods are considered [24].

Our results confirm that report of multiple distressing somatic symptoms constitutes a syndrome, the co-occurrence of symptoms being much more frequent than would be expected by chance. However, there was no clear dichotomy between people with and without somatising tendency. Thus, the strength of associations, both with multi-site pain and with sickness absence for non-musculoskeletal reasons, increased progressively with the number of symptoms reported, at least up to five. Because these associations were cross-sectional, they cannot necessarily be interpreted as causal, although longitudinal studies have indicated that people who complain of common somatic symptoms are more likely to develop multisite musculoskeletal pain subsequently [19,20]. We also found that all seven of the symptoms investigated contributed to the measurement of somatising tendency, with smaller attributable fractions for multi-site pain and non-musculoskeletal sickness absence when any one of the symptoms was disregarded. However, the differences in PAFs were generally small, and if resources were limited, it is likely that little would be lost if any one of the seven symptoms were omitted from the question set.

Follow-up of participants after approximately 14 months demonstrated that levels of somatising tendency were fairly stable within individuals over that timescale, and the observation that this occurred despite changes in the specific symptoms reported is evidence that the consistency reflects a continuing general predisposition to be aware of and report physical symptoms, rather than persistence of specific underlying disease. A similar pattern has been found in earlier longitudinal studies [24]. It is notable, however, that a small minority of participants exhibited major changes in their degree of somatising tendency, suggesting that it is not entirely a fixed trait, and raising the possibility that it might in some cases be amenable to intervention. Another possibility is that these large changes reflected the development or resolution of co-morbidity.

The higher frequency of somatic symptoms among women than men accords with other studies [25–27]. It has been postulated that the imbalance may reflect innate differences in somatic and visceral perception; differences in symptom labelling, description and reporting; or a greater willingness of women to acknowledge and disclose discomfort [25]. It could also arise from a higher prevalence of depression in women.

Somatisation has also been reported to occur more commonly at older ages [23]. We too found a positive relationship to age in women, although in men, the prevalence of somatic symptoms was highest at younger ages. Because our analysis was cross-sectional, it was not possible to distinguish effects of age from trends across birth cohorts. However, the higher prevalence of hot or cold flushes among older women may have been a physiological effect of age.

The large differences between occupational groups and countries in the prevalence and degree of somatising tendency were apparent even after adjustment for differences in sex and age. As already discussed, the variation may have been, at least in part, a linguistic artefact. However, earlier research using different methods has also indicated unusually high rates of somatisation in South America [23]. In that study, there was no evidence that somatising patients from South America had a lower prevalence of co-occurring depression or generalised anxiety disorder, which suggests that their somatisation was not a manifestation of occult mental illness. Perhaps more likely is a culturally determined difference in the perception of bodily sensations and the importance that is attached to them, or in willingness to report them when they occur. There was also variation between countries in the relative frequency of specific somatic symptoms, but to a lesser extent.

Somatisation has previously been linked with an absence of formal education [23], but after allowance for sex, age and occupational group, we found no relationship to level of education. This may have been because within occupational groups there was too little heterogeneity for an effect to be discernible. We did, however, find a weak association with smoking, which is consistent with an earlier study in Finnish adolescents [28].

In summary, our study supports the use of questions from the Brief Symptom Inventory as a method for measuring tendency to somatise, each of the seven questions contributing to its assessment. The findings indicate that somatising tendency should be regarded as a quantifiable characteristic that exhibits an exposure-response relationship in its association with other health measures, and appears to be fairly stable over an interval of approximately one year, although the specific symptoms that individuals report frequently vary over time. It is more common in women than in men, especially at older ages, and its prevalence varies between countries with higher rates in South and Central America.

Given its potential to explain differences in disability and in economically important outcomes such as sickness absence from work, there is a need to understand further what drives somatising tendency, and whether and how it might be modified at a population level. There is evidence, for example, that it tracks across generations [29], and it may be a trait which is acquired early in life. Thus, there is a need for further research to establish how it evolves at younger ages, what influences its development, and also how constant it remains over longer follow-up periods.

## Supporting Information

S1 Appendix Committees which provided ethical approval for the CUPID study. (DOCX)

S1 Dataset Supporting Dataset. (DTA)

S1 Metadata Metadata. (XLSX)
