# Supplementary material for: Descriptive Epidemiology of Somatising Tendency: Findings from the CUPID Study
Source: PLoS One. 2016 Apr 29;11(4):e0153748. doi: 10.1371/journal.pone.0153748 (PMC4851348; doi:10.1371/journal.pone.0153748)
Supplement: S1 Appendix — (DOCX) [file pone.0153748.s001.docx]

**APPENDIX A: COMMITTEES WHICH PROVIDED ETHICAL APPROVAL FOR THE CUPID STUDY**

Brazil: National Committee for Ethics in Research and Ethics Committee of University Hospital of University of Sao Paolo

Ecuador: Ethical Committee of Biomedicine, Central University of Ecuador

Colombia: Ethics Committee of the School of Medicine, Pontificia Universidad Javeriana, Bogotá, Colombia

Costa Rica: Ethics Committee of the Universidad Nacional in Costa Rica

Nicaragua: Ethics Committee for Biomedical research of the Universidad Nacional Autonoma de Nicaragua

UK: National Research Ethics Service Committee South Central - Berkshire

Spain Parc Salut Mar Ethics Committee of Barcelona

Italy: Institutional Review Boards, Fondazione IRCCS Ca’ Granda – Ospedale Maggiore Policlinico (Milan) and Ospedale di Circolo Fondazione Macchi (Varese)

Greece: Scientific Board Committee of the University Hospital of Heraklion

Estonia: Ethics Review Committee on Human Research, University of Tartu

Lebanon: Institutional Review Board, American University of Beirut

Iran: Research Committee of Shahroud University of Medical Sciences

Pakistan: Ethical Review Committee of Aga Khan University

Sri Lanka: Ethical review Committee, Faculty of medical Sciences, University of Sri Jayawardenepura

Japan: University of Tokyo Ethics Committee

South Africa: University of Witwatersrand Ethics Committee for Human Subjects

Australia: Monash University Human Research Ethics Committee and the Alfred Ethics Committee

New Zealand: New Zealand Multi-region Ethics Committee
